# Effect of Occlusal Scheme and Bone‐Level Implant Number and Position on Stress Distribution in Kennedy Class II Implant‐Assisted Removable Partial Dentures: A 3D Finite Element Analysis

**DOI:** 10.1002/cre2.70078

**Published:** 2025-01-20

**Authors:** Solmaz Barati, Safoura Ghodsi, Somayeh Zeighami

**Affiliations:** ^1^ Department of Prosthodontics, School of Dentistry Tehran University of Medical Sciences Tehran Iran; ^2^ Dental Research Center, Dentistry Research Institue and Department of Prosthodontics, School of Dentistry Tehran University of Medical Sciences Tehran Iran

**Keywords:** dental occlusion, finite element analysis, implant‐supported denture, removable partial denture

## Abstract

**Objectives:**

To assess the effect of occlusion and implant number/position on stress distribution in Kennedy Class II implant‐assisted removable partial denture (IARPD).

**Materials and Methods:**

IARPDs were designed in six models: with one implant (bone level with a platform of 4 mm and length of 10 mm) at the site of (I) canine, (II) between first and second premolars, (III) first molar, (IV) second molar, or two implants at the sites of (V) canine‐first molar, and (VI) canine‐second molar. A conventional RPD served as control. Loads were applied according to the group function (GF) (500N load was applied to the left canine/premolar/molar teeth in the ratio of 1:1:2) or canine guidance (CG) (125N load was to the canine tooth) occlusions. Maximum displacement and Von Mises Stress in different components were analyzed by finite element analysis (FEA).

**Results:**

The control model showed the highest displacement followed by the IARPD with a canine implant in both occlusal schemes. In GF, the maximum and minimum jaw stress were recorded in IARPDs with canine implants (16.45 MPa) and canine‐first molar implants (13.47 MPa), respectively. In CG, the maximum and minimum jaw stress was recorded in IARPD with first/second premolar implant (15.91 MPa) and canine‐first molar implants (12.38 MPa), respectively. The highest stress in resin, framework, and implant(s) was noted in IARPD with canine implant in both schemes. The lowest stress in the implant(s) was recorded in IARPD with canine‐second molar implants in GP and IARPD with canine‐first molar implants in CG.

**Conclusion:**

Dental implants reduced the total displacement of IARPDs, increased stress in mechanical components, and did not affect stress distribution in biological components. Insertion of two implants decreased implant stress. The GP scheme caused greater stress on mechanical components.

## 1 Introduction

Epidemiological studies on partial edentulism and tooth loss vary considerably in terms of prevalence in different countries and various regions of a country (Fayad, Baig, and Alrawaili 2016).

The numbering of the Kennedy classification system (I, II, III, and IV) for partially edentulous dental arches is partly based on the prevalence in the population, indicating that Class I arches are the most common and Class IV arches are the least common (Phoenix, Cagna, and DeFreest (2008). Stewart's clinical removable partial prosthodontics (4th ed.: Quintessence Publishing Co Inc.) In two studies on the prevalence of partial edentulism patients, class II Kennedy edentulism was ranked second (Fayad, Baig, and Alrawaili 2016) and third (Al‐Angari et al. 2021).

A removable partial denture (RPD) is a suitable treatment option for partially edentulous patients. However, the continuation of the resorption process in the residual ridge over time decreases the stability and support of RPD and causes patient discomfort and dissatisfaction (Shue, Miron, and Yufeng 2016).

Conventional free‐end RPDs have a complex load distribution pattern due to differences in flexibility and viscoelastic properties of the soft tissue and teeth (Shahmiri et al. 2013). Such disharmony in tissue response and load distribution would result in torquing forces applied to the abutment teeth and rotational movements of the free‐end RPD, which would eventually cause damage to the abutment teeth, soft tissue, and bone (Fayaz et al. 2015). Patients with free‐end partially edentulous situations (Kennedy Class I and II) have more problems in terms of retention, support, and stability compared with tooth‐bound edentulism. In Kennedy Class II, the situation is even more challenging since a higher number of retentive clasps are required in the dentate quadrant to achieve cross‐arch stability (Allen 2003). This asymmetrical edentulism would adversely affect patient satisfaction especially when opposed by a completely dentate arch. The canine tooth has a prominent role in load distribution due to its strategic position in the dental arch, large crown, and relatively large periodontal ligament surface. Thus, the loss of canine tooth further aggravates the condition in partially edentulous arches (Allen 2003).

The complications of free‐end RPDs led to the increasing use of dental implants as a solution to decrease biological and biomechanical complications (Shahmiri et al. 2013). Dental implants can participate in the support, stability, and retention of free‐end RPDs (Ortiz‐Puigpelat et al. 2019). Studies on IARPDs for different classes of partial edentulism have pointed to the significant effect of location, position, and number of dental implants on stress distribution in teeth and adjacent tissues that reported conflicting results (Cunha et al. 2008; Eom et al. 2017; Jia‐Mahasap et al. 2022; Mahshid et al. 2014; Matsudate et al. 2016; Memari et al. 2014; Mijiritsky et al. 2015; Ortiz‐Puigpelat et al. 2019; Verri et al. 2011). The occlusal scheme is another important factor affecting stress distribution in both dentate and partially edentulous patients. The possibility of determining the best occlusal scheme for reconstructing implant‐supported prostheses still seems impossible due to insufficient scientific evidence (Gomes et al. 2021).

Finite element analysis (FEA) has long been used for the prediction of the biological behavior of dental implants (Eom et al. 2017). The majority of relevant FEA studies evaluated Kennedy Class I IARPDs (Mahshid et al. 2014; Memari et al. 2014; Ortiz‐Puigpelat et al. 2019; Shahmiri et al. 2013, 2014; Shahmiri and Atieh 2010). A literature search by the authors revealed no studies on the effect of different occlusal schemes on the pattern of stress distribution in biological (bone, soft tissue, and tooth) and mechanical (resin, framework, and implant) components in Kennedy Class II IARPDs. Therefore, this study aimed to assess the effect of the occlusal scheme and the number and position of dental implants on stress distribution patterns in Kennedy Class II IARPDs using FEA. The null hypothesis was that the implant placement, implant position/number, and occlusion have no significant effect on the total displacement of IARPD or stress distribution pattern in biological and mechanical components.

## 2 Materials and Methods

The study was approved by the ethics committee of Tehran University of Medical Sciences (IR.TUMS.DENTISTRY.REC.1400.174).

All methods were carried out in accordance with relevant guidelines and regulations.

### 2.1 Study Model

The study cast was fabricated from a rubbery mold of the mandible (Frasaco GmbH, Germany) by using plaster dental stone (Type IV Synthetic Die & Model Stone, GC FUJIROCK EP), and was then modified such that the left‐side mandibular canine, premolars, and molars were removed to create a unilateral partially edentulous ridge. A surveyor (Degussa Frasgerat F1 Milling Machine) was used to parallelize the position of the cast to the horizontal line and identify the height of contour for the retentive and reciprocating arms. Corrections and recontouring were performed, and a #10 gauge was used to determine the finishing location of the retentive arms. The desired design was then drawn on the cast, which included a lingual bar major connector, I bar, cingulum rest (cingulum rests are rarely prepared on incisor teeth and are only indicated when the canine tooth is absent), and proximal plate at the distal of left lateral incisor, and an embrasure clasp with retentive arms at the buccal and reciprocating arms at the lingual of the right first and second molars. On the first premolar, a minor connector and mesio‐occlusal rest as an indirect retainer and a retentive arm on the buccal surface and reciprocal arm on the lingual surface were designed. Design and preparation were according to Stewart's concept (Phoenix, Cagna, and DeFreest 2008).

### 2.2 Designing of RPD by the Software

**Figure 1:**
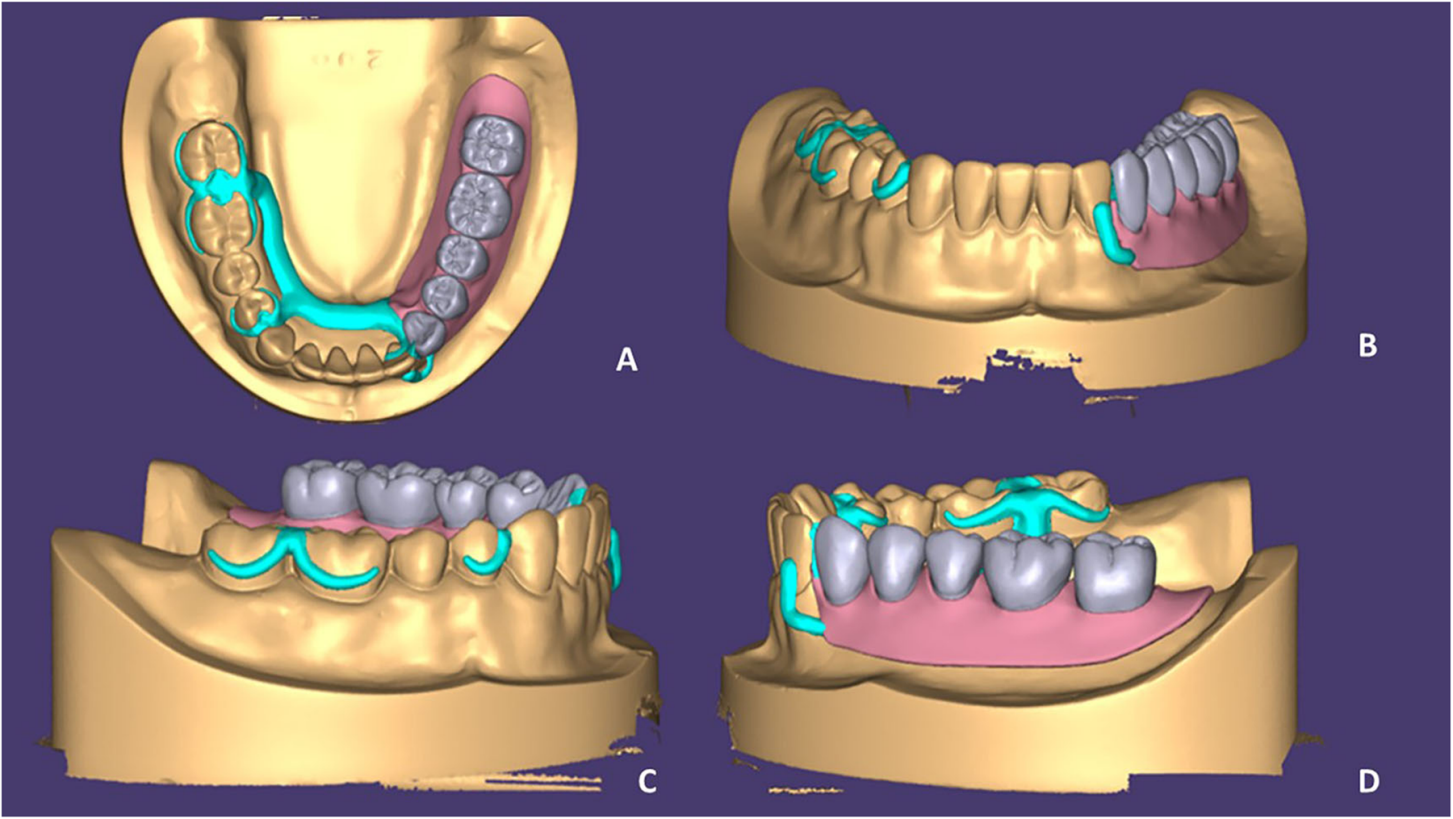
Digital design of conventional RPD. (A) Occlusal view, (B) frontal view, (C) right side view, and (D) left side view.

The study cast was scanned using a laboratory scanner (Ceramill Map 400; Amann Girrbach AG), and the scan file was transferred to Exocad/Partial CAD 3.0 Galway. To design the RPD framework in the software, first, the lost teeth were digitally mounted in the edentulous region with proper shape and number (from the left canine to the second molar) to obtain data regarding the design including the extension width of the framework. The framework was designed as desired and some modifications were also made manually. The lingual bar was designed with a semi‐pear‐shaped cross‐sectional design and standard dimensions. The base areas were designed by considering a 1 mm relief for the acrylic resin. Other areas such as the internal and external finish lines were also determined. Finally, a conventional RPD framework was designed (Figure 1). The acrylic resin (beneath the base and at the flanges) and teeth were also added to the RPD framework.

**Figure 2:**
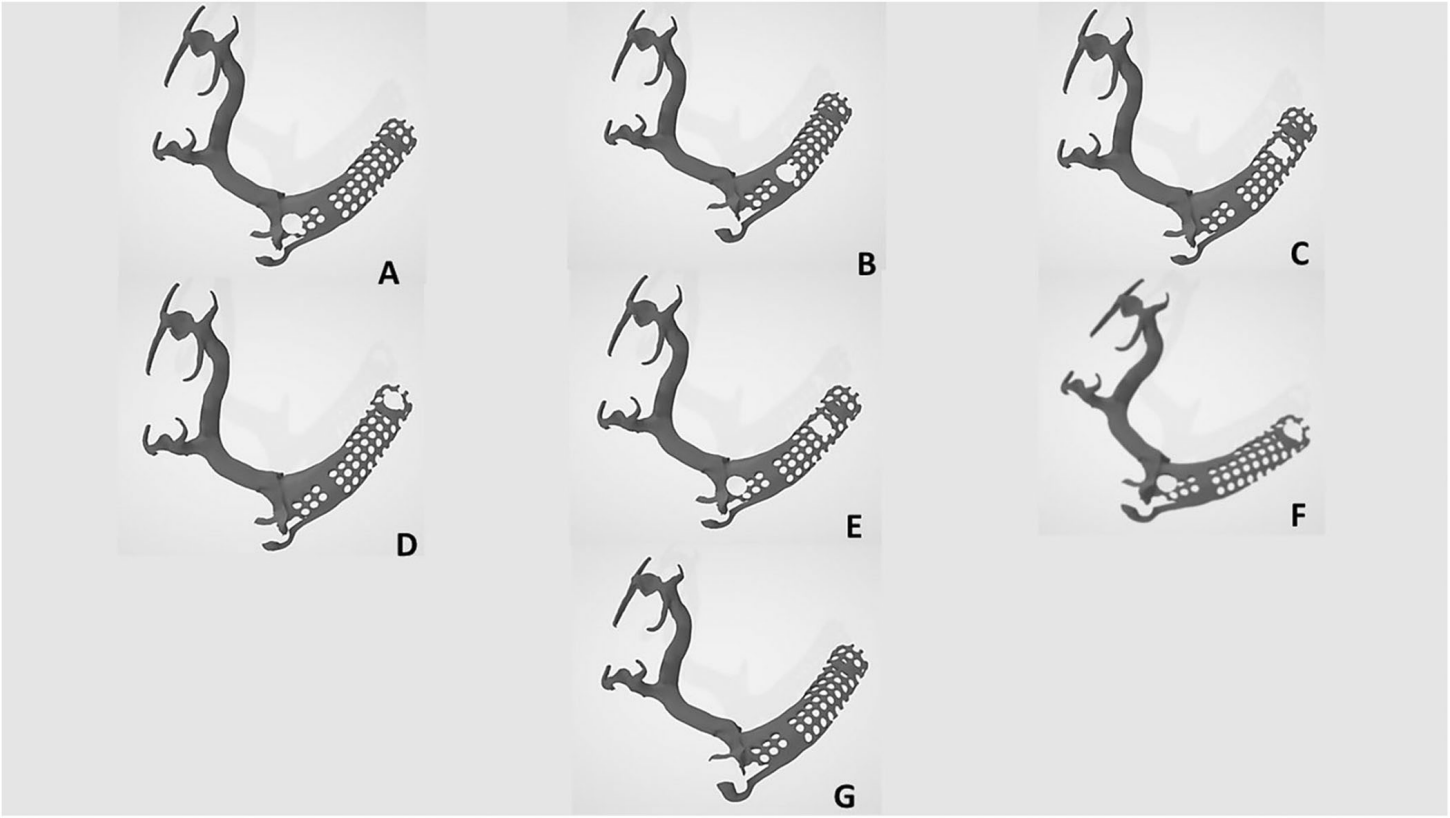
Digitally designed frameworks; (A) IARPD with one dental implant at the site of canine; (B) IARPD with one dental implant placed between first and second premolars; (C) IARPD with one dental implant placed at the site of first molar; (D) IARPD with one dental implant placed at the site of second molar; (E) IARPD with two dental implants placed at the sites of canine and first molar teeth; (F) IARPD with two dental implants placed at the sites of canine and second molar teeth; (G) conventional RPD.

The framework of IARPDs was designed by mesh construction with the opening in the implant position(s) to the diameter of 4.6 mm. In this design, the attachment housing contacts the acrylic resin instead of metal, resulting in better stress distribution. Also, this design enables the correction of errors and relining in the clinical setting. Accordingly, six different frameworks were designed (Figure 2) as follows: (I) IARPD with one dental implant at the site of the canine, (II) IARPD with one implant placed between the first and second premolars, (III) IARPD with one implant placed at the site of first molar, (IV) IARPD with one implant placed at the site of second molar, (V) IARPD with two implants at the sites of canine and first molar teeth, and (VI) IARPD with two implants at the sites of canine and second molar teeth (Misch 2015; Ortiz‐Puigpelat et al. 2019; Phoenix, Cagna, and DeFreest 2008; Shue, Miron, and Yufeng 2016; Verri et al. 2011).

### 2.3 Digital Modeling

Digital modeling was performed in SolidWorks software version 14 (Dassault Systems SolidWorks Corp., Waltham, MA) followed by a simplification and decrease in the mandible size for faster analysis. The designing of implant components was performed as well. The selected implant (SuperLine implants, Dentium) had a 4 mm diameter and 10 mm length (Eom et al. 2017). The attachment of choice was the Locator (Zest Anchors LLC, Carlabad, CA), which is widely used as an anchoring component due to its ease of use, repair, and replacement (Oh, Oh, and Park 2016). Also, all design data of RPD were transferred from Exocad/Partial CAD 3.0 Galway to SolidWorks software in the format of STL (stereolithography) files. Considering the two occlusal schemes of group function (GP) and canine guidance (CG), a total of 14 models (with the inclusion of conventional RPD as control) were designed and evaluated.

### 2.4 FEA

**Figure 3:**
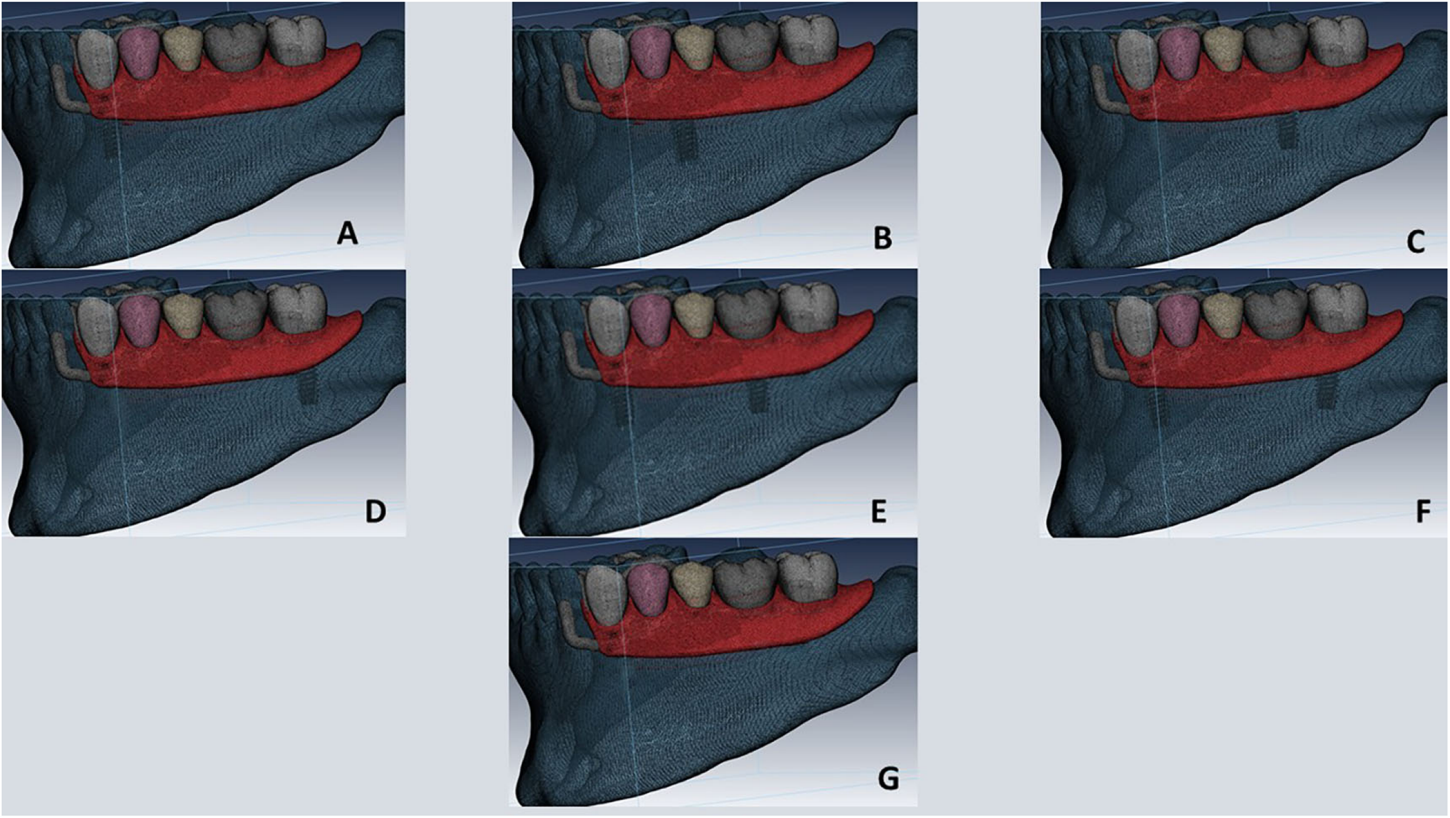
Study models meshing; (A) IARPD with one dental implant at the site of canine; (B) IARPD with one dental implant placed between first and second premolars; (C) IARPD with one dental implant placed at the site of first molar; (D) IARPD with one dental implant placed at the site of second molar; (E) IARPD with two dental implants placed at the sites of canine and first molar teeth; (F) IARPD with two dental implants placed at the sites of canine and second molar teeth; (G) conventional RPD.

**Figure 4:**
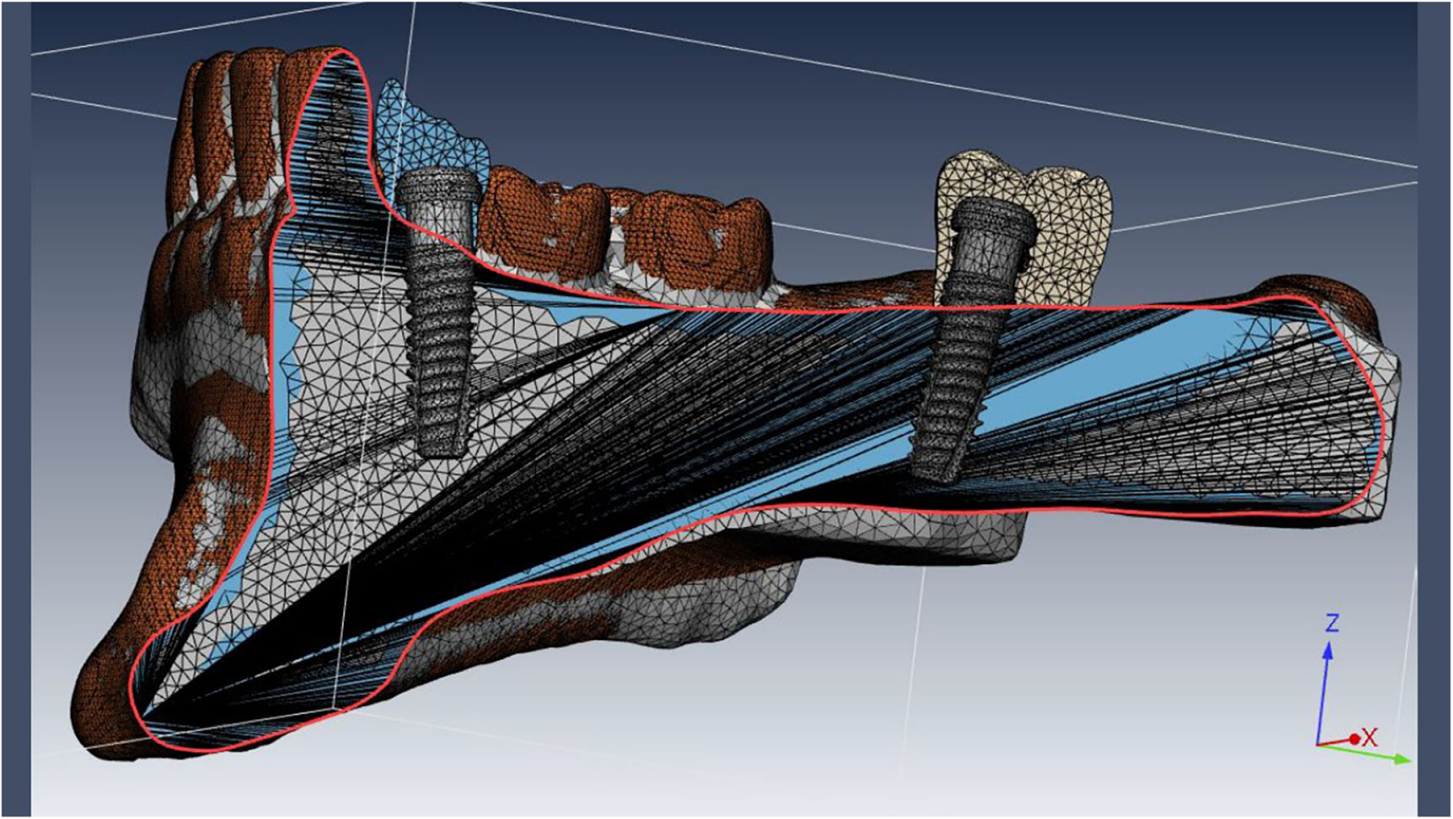
Cross‐section of mesh elements (C3D4 or tetrahedral) and boundary conditions in IARPD with two dental implants placed at the sites of canine and second molar teeth.

A native meshing was first performed. Next, each part instance in the assembly module was created independently. Final meshing was subsequently performed with tetrahedral (C3D4) elements. The largest element was 0.8 mm, and the smallest element was 0.5 mm. The total number of elements was 290.536 and the total number of nodes was 248.394 (Figures 3 and 4).

The external surface of implant threads was tied to the internal surface of bone. The boundary conditions were applied in the Load section of Abaqus software. The BC > Encastre command was used to limit both sides of the jaw. A dental implant was defined as a rigid body (zero deformation). A reference point was also defined for the implant to apply boundary conditions and loads to this point. All structures were considered isotropic, and Young's modulus (MPa) and Poisson's ratio were defined for them (Table 1).

**Table 1:** Physical characteristics of the components (Ortiz‐Puigpelat et al. 2019).

Material	Young's modulus (GPa)	Poisson's coefficient
Jawbone	4.4525	0.3
Teeth	18.6	0.31
Alveolar mucosa	0.68	0.45
Titanium Implant		
Locator retainer	103.4	0.35
Chrome‐cobalt metal structure	206.9	0.33
Acrylic resin	8.3	0.28
Nylon retentive part of the locator	2.4	0.39

IARPD of the mandible was considered against natural teeth. Thus, to simulate the masticatory forces in the GF scheme, 500N load was applied to the left canine/premolar/molar teeth in a ratio of 1:1:2 at a 30‐degree angle from the buccal direction. In the CG scheme, 125N load was applied to the canine tooth at a 30‐degree angle from the buccal direction (Göre and Evlioğlu 2014). FEA was then performed. Total deformation and the maximum von Mises stress values were reported for all models.

## 3 Results

Tables 2 and 3 show the total deformation and maximum von Mises stress values in the GF and CG occlusal schemes, respectively.

**Table 2:** Total deformation (mm) and maximum von Mises stress (MPa) values in the GF occlusal scheme.

	Canine implant	First/second premolar implant	First molar implant	Second molar implant	Canine and first molar implants	Canine and second molar implants	Control
Total deformation	0.084	0.075	0.079	0.078	0.075	0.071	0.1
Stress in the jaw	16.45	14.70	14.82	14.76	13.47	14.81	14.11
Stress in soft tissue	3.6	3.3	3.4	3.1	3.2	3.3	2.8
Stress in framework	171.2	159.7	164.9	167.8	154.3	158.8	122.7
Stress in resin	34.74	31.82	32.26	32.14	31.24	33.19	31.11
Stress in implant(s)	174.23	138.1	155.4	153.7	139.6	137.6	—
Stress in abutment tooth #32	17.24	16.72	16.89		16.87	16.94	15.98
Stress in abutment tooth #44	15.85	14.84	14.87	14.91	15.23	14.98	14.73
Stress in abutment teeth #46 and #47	14.26	13.76	13.83	13.63	13.29	13.85	13.71

**Table 3:** Total deformation (mm) and maximum von Mises stress (MPa) values in the CG occlusal scheme.

	Canine implant	First/second premolar implant	First molar implant	Second molar implant	Canine and first molar implants	Canine and second molar implants	Control
Total deformation	0.095	0.073	0.076	0.075	0.072	0.070	0.12
Stress in the jaw	15.63	15.91	15.42	14.94	12.38	14.23	14.82
Stress in soft tissue	3.6	3.8	3.3	2.9	3.1	3.7	2.9
Stress in framework	169.4	124.7	124.6	117.9	127.2	137.4	128.6
Stress in resin	35.15	27.21	31.58	25.42	29.54	27.19	24.32
Stress in implant(s)	173.46	126.47	154.71	154.29	117.25	139.2	—
Stress in abutment tooth #32	16.24	16.53	15.62	17.43	17.15	15.94	17.29
Stress in abutment tooth #44	14.73	15.01	15.04	14.96	14.85	14.22	14.98
Stress in abutment teeth #46 and #47	13.37	13.52	13.74	13.26	13.97	13.85	13.27

### 3.1 Total Displacement

In the GP, the highest displacement occurred in the control model (0.1 mm) followed by the IARPD with canine implant (0.95 mm). In the CG, the highest displacement occurred in the control model (0.12 mm) followed by the IARPD with canine implant (0.084 mm). Displacement was almost the same in the other models both in GF and CG.

### 3.2 Jaw Stress Pattern

In the GP, the highest and the lowest stress value in the jaw was recorded in IARPD with canine implant (16.45 MPa) and canine and first molar implant models (13.47 MPa), respectively. In the CG, the highest and lowest stress value in the jaw was recorded in IARPD with First/second premolar implant (15.91 MPa) and canine‐first molar implants (12.38 MPa), respectively. The stress values were almost the same in other models both in GF and CG.

### 3.3 Soft Tissue Stress Pattern

In both the GF and CG, soft tissue stress was almost the same in all IARPD models, and the lowest stress was noted in the control model in both occlusal schemes (2.8 MPa for the GF and 2.9 MPa for the CG scheme).

### 3.4 Metal Framework Stress Pattern

In the GF, the highest stress value was recorded in IARPD with canine implant (171.2 MPa), and the lowest was recorded in the control model (122.7 MPa). In the CG, the highest stress value was recorded in IARPD with canine implant (169.4 MPa), and the lowest was recorded in the IARPD with second molar implant (117.9 MPa).

### 3.5 Resin Base Stress Pattern

In the GF, the highest stress value was recorded in IARPD with canine implant (34.74 MPa). The stress distribution pattern was almost the same for other models. In the CG, the highest stress value was recorded in IARPD with canine implant (35.15 MPa), and the lowest stress value was recorded in the control model (24.32 MPa).

### 3.6 Last Abutment Tooth (#32) Stress Pattern

Stress on tooth #32 was almost the same in all models for both occlusal schemes.

### 3.7 Implant(s) Stress Pattern

**Figure 5:**
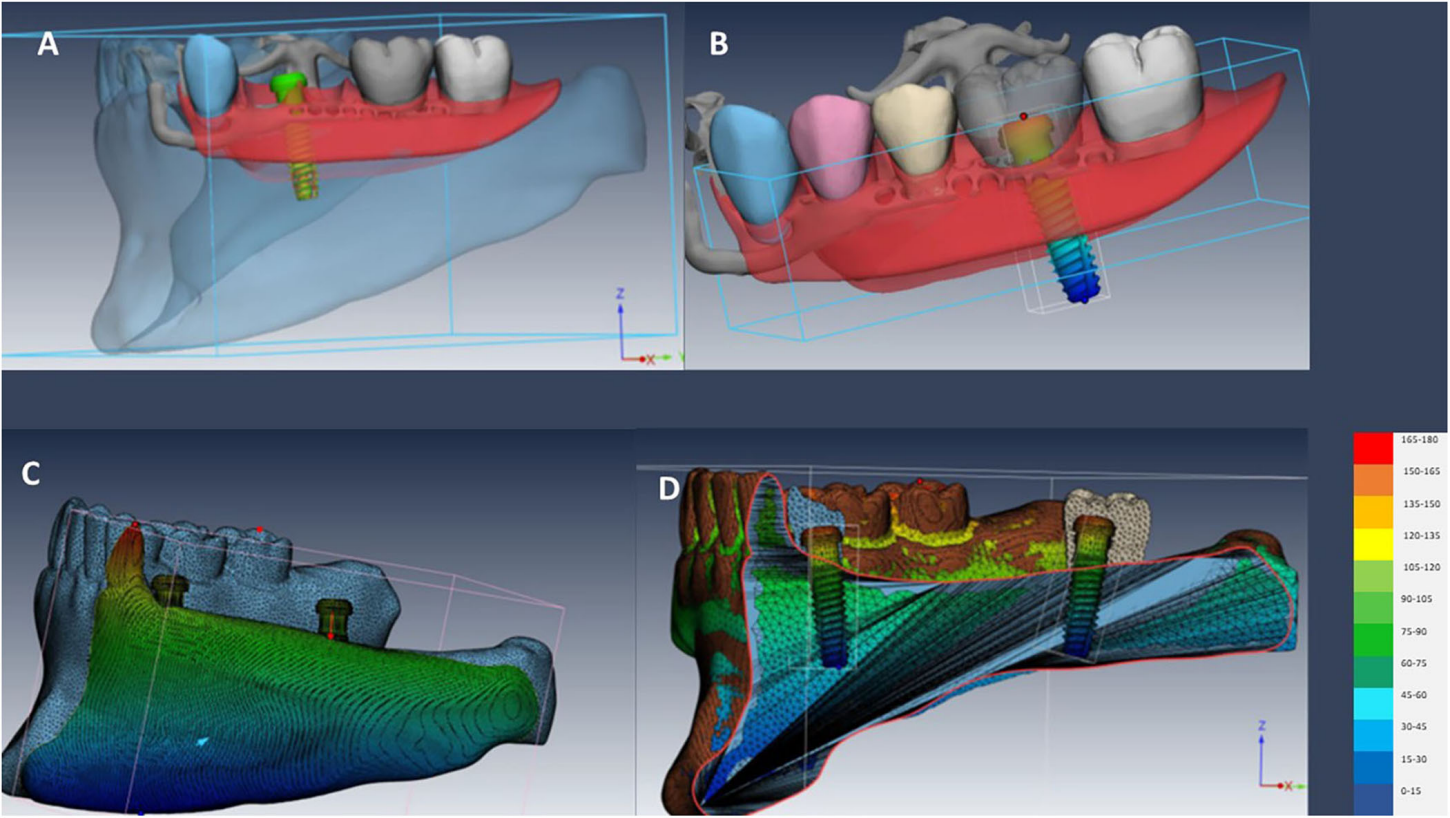
Stress maps on implants, bone, and locators in some study models; (A) IARPD with one dental implant placed between first and second premolars and CG occlusal scheme; (B) IARPD with one dental implant placed at the site of first molar and CG occlusal scheme; (C) IARPD with two dental implants placed at the sites of canine and first molar teeth and GF occlusal scheme; (D) IARPD with two dental implants placed at the sites of canine and second molar teeth and GF occlusal scheme.

**Figure 6:**
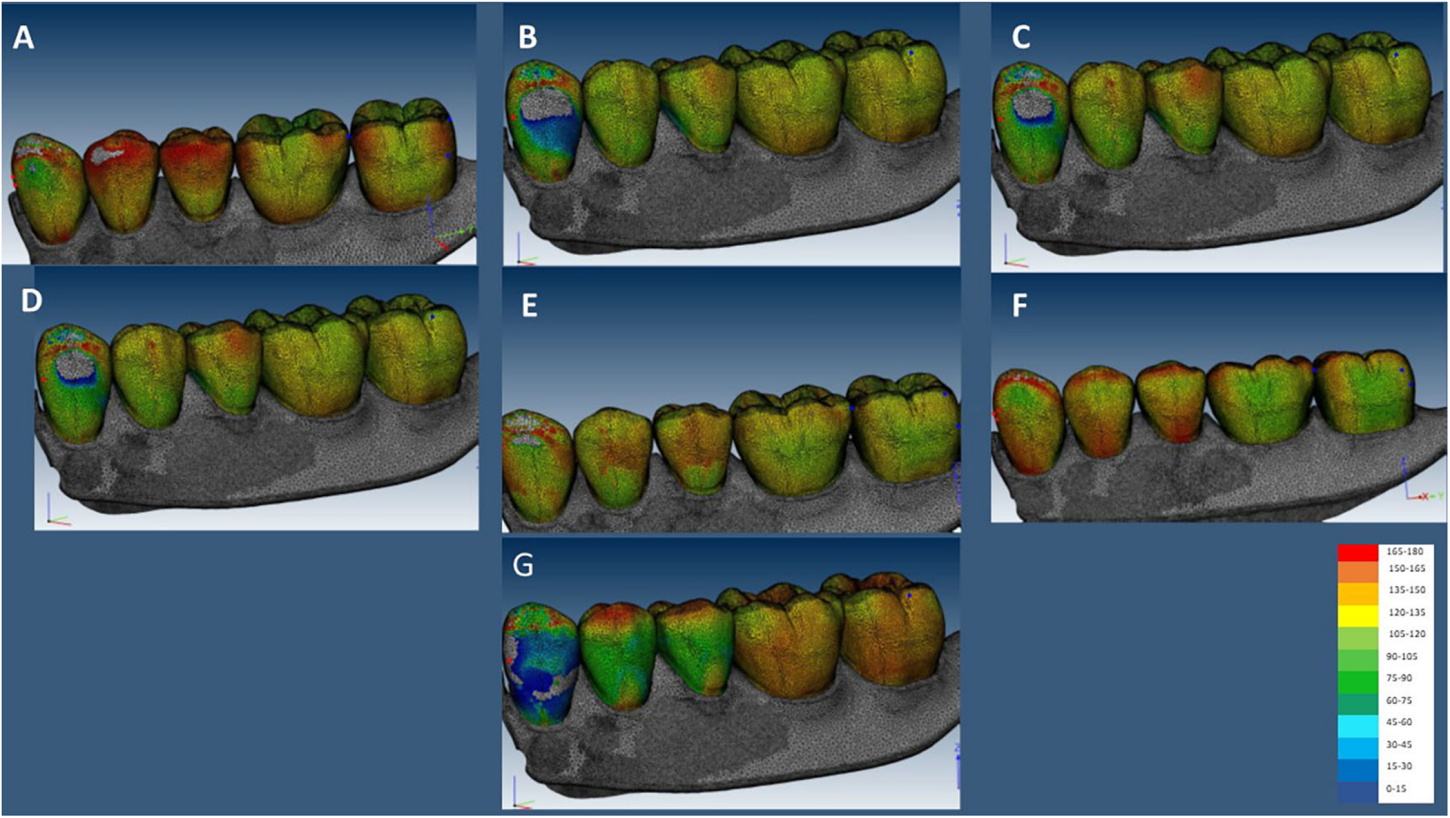
Stress applied to resin teeth in the CG occlusal scheme; (A) IARPD with one dental implant at the site of canine; (B) IARPD with one dental implant placed between first and second premolars; (C) IARPD with one dental implant placed at the site of first molar; (D) IARPD with one dental implant placed at the site of second molar; (E) IARPD with two dental implants placed at the sites of canine and first molar teeth; (F) IARPD with two dental implants placed at the sites of canine and second molar teeth; (G) conventional RPD.

**Figure 7:**
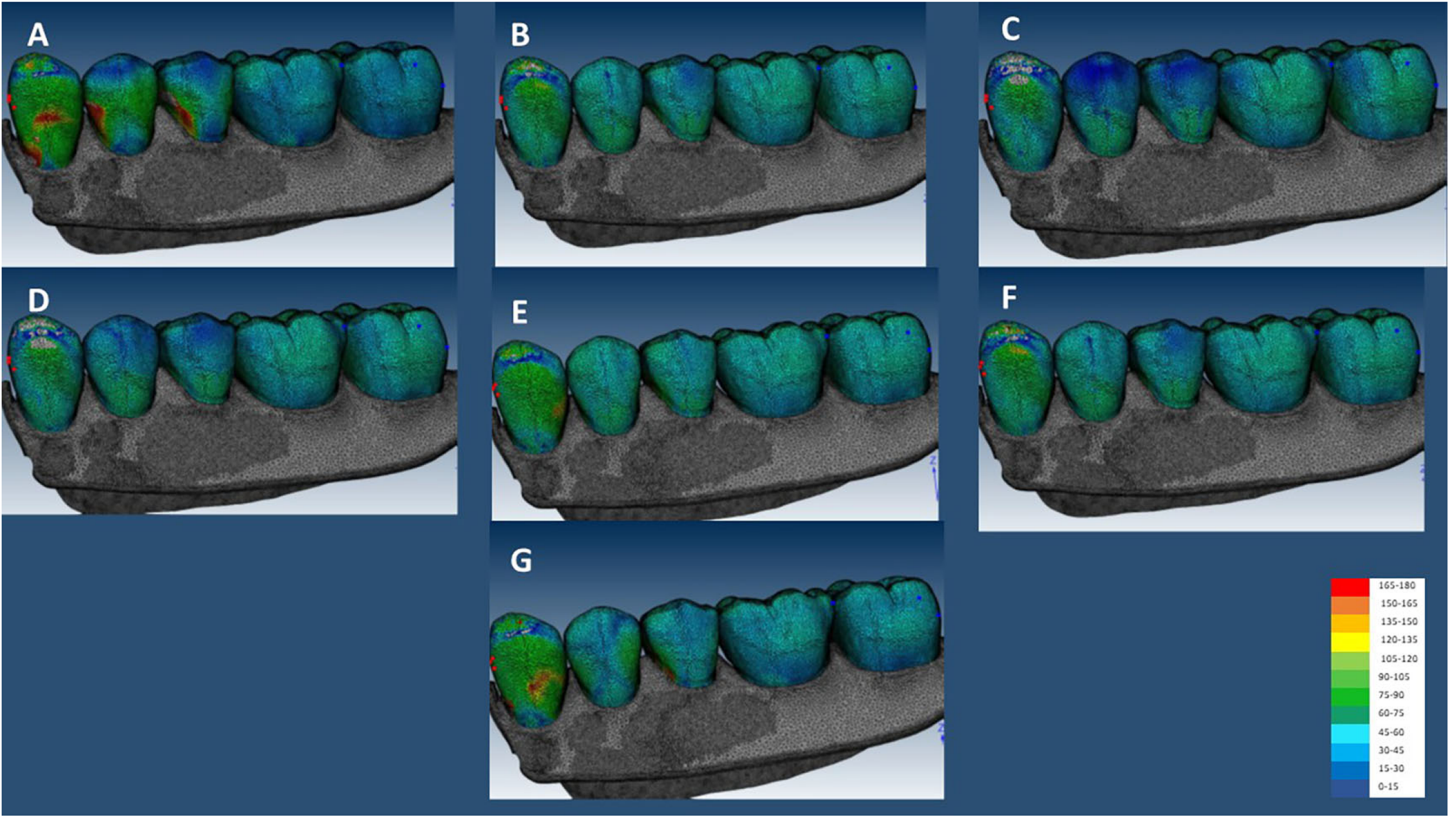
Stress applied to resin teeth in the GF occlusal scheme; (A) IARPD with one dental implant at the site of canine; (B) IARPD with one dental implant placed between first and second premolars; (C) IARPD with one dental implant placed at the site of first molar; (D) IARPD with one dental implant placed at the site of second molar; (E) IARPD with two dental implants placed at the sites of canine and first molar teeth; (F) IARPD with two dental implants placed at the sites of canine and second molar teeth; (G) conventional RPD.

In the GF, the highest stress value in implant was recorded in IARPD with canine implant (174.23 MPa), and the lowest was recorded in IARPD with two implants at the sites of canine and second molar teeth (137.6 MPa). In the CG, the highest stress value was recorded in IARPD with canine implant (173.46 MPa), and the lowest was recorded in IARPD with two implants at the sites of canine and first molar teeth (117.25 MPa) (Figures 5, 6, 7).

## 4 Discussion

The purpose of this study was to assess the effect of occlusion and dental implant number/position on stress distribution in Kennedy Class II IARPDs using FEA. The null hypothesis of the study was that implant placement, implant position/number, and occlusion would have no significant effect on the total displacement of IARPD or stress distribution pattern in biological and mechanical components.

The results showed that the part of the null hypothesis regarding no significant effect of implant placement on the total displacement of RPD in both occlusal schemes was rejected since implant placement decreased the total displacement of IARPDs. Previous studies have also confirmed that the retention and support provided by dental implants in RPDs would prevent rotation around the fulcrum axis (Bassetti, Bassetti, and Kuttenberger 2018; Cunha et al. 2008; Eom et al. 2017; Ichikawa et al. 2023).

The part of the null hypothesis regarding no significant effect of implant number on the total displacement of IARPDs in both occlusal schemes was confirmed since the results showed that placement of two implants at the canine and molar sites decreased the displacement compared with the control model but had no difference with RPDs supported by one implant. In contrast, Eom et al. (2017) reported a significant reduction in RPD displacement in the placement of two implants compared with one implant. It appears that placement of one implant would probably decrease the displacement of RPD; however, in cases with severe ridge resorption or bruxers, two implants (one in the anterior and the other one in the posterior) need to be placed.

The part of the null hypothesis regarding no significant effect of implant position on the total displacement of RPDs in the two occlusal schemes was partially rejected since in both occlusal schemes, maximum displacement was noted in IARPD with canine implant and displacement was almost the same in all other models. Evidence shows that in Kennedy Class I and II partial edentulism, implant placement in the posterior region causes a greater reduction in displacement than implant insertion in the anterior region (Cunha et al. 2008; Memari et al. 2014; Oh, Oh, and Park 2016; Ortiz‐Puigpelat et al. 2019). Nonetheless, it should be noted that, unlike the previous studies, canine tooth was not present in models of the current study. Thus, the present results cannot be accurately compared with the previous findings.

The part of the null hypothesis regarding the insignificant effect of occlusion on RPD total displacement was partially rejected because the displacement of the conventional RPD and IARPD with canine implant was greater in the CG than in the GF scheme. Other IARPDs had no noticeable difference in the two occlusal schemes. Greater displacement in the CG scheme may be due to the fact that conventional RPD and IARPD are more susceptible to displacement upon application of lateral forces to canine tooth, and GF may be a better scheme for such restorations.

The part of the null hypothesis regarding the insignificant effect of implant placement on jaw stress in the two occlusal schemes was partially rejected due to considerably higher stress distribution in the jaw in IARPD with canine implant and GF scheme. Stress analysis of the mandible in a study by Ortiz‐Puigpelat et al. (2019) showed that implant placement, irrespective of its location, caused no change in stress distribution pattern in the jaw.

The null hypothesis regarding the insignificant effect of implant number on jaw stress in the two occlusal schemes was also partially rejected because jaw stress was lower in IARPDs with dental implants placed at the sites of canine and first molar teeth but IARPD with canine and second molar implants had no significant difference with other models in this regard. Jia‐Mahasap et al. (2022) revealed that increasing the number of implants from one to two decreased jaw stress. Therefore, if two implants are to be placed, canine and first molar sites are recommended.

The null hypothesis regarding the insignificant effect of implant position on jaw stress in the two occlusal schemes was partially rejected since stress level was the highest in IARPD with canine implant and the lowest in IARPD with canine and first molar implants. It has been reported that implant placement at the site of the first molar results in better stress distribution in the mandible (Memari et al. 2014; Ortiz‐Puigpelat et al. 2019). However, Jia‐Mahasap et al. (2022) found no significant difference in jaw stress in IARPDs with one implant placed at different locations.

In IARPD with canine implant and IARPD with canine and first molar implants, jaw stress was greater in GF while in IARPD with one dental implant placed between the first and second premolars or second molar site, the jaw stress was greater in the CG scheme. Therefore, part of the null hypothesis regarding the insignificant effect of the occlusal scheme on jaw stress was partially rejected. Due to the diversity of results in this regard, no definite conclusion can be drawn in this respect.

The part of the null hypothesis regarding the insignificant effect of implant insertion on soft tissue stress was confirmed in both occlusal schemes since no significant difference was found in soft tissue stress between conventional RPD and IARPDs. This finding was in contrast to studies that reported a reduction in stress in biological components following implant placement in RPDs (Cunha et al. 2008; Memari et al. 2014; Ortiz‐Puigpelat et al. 2019; Pellizzer et al. 2010; Verri et al. 2011). The number of implants had no significant effect on soft tissue stress in the two occlusal schemes; thus, the null hypothesis in this regard was confirmed. This finding was in agreement with the results of Ortiz‐Puigpelat et al. (2019). Implant position had no significant effect on soft tissue stress in the two occlusal schemes; therefore, this hypothesis was also confirmed. However, Ortiz‐Puigpelat et al. (2019) indicated that implant insertion close to the abutment tooth increased soft tissue stress while posterior implant insertion resulted in better stress distribution in the soft tissue (Cunha et al. 2008; Pellizzer et al. 2010). The type of occlusion had no significant effect on soft tissue stress either, confirming the null hypothesis in this regard.

Implant insertion increased metal framework stress in both occlusal schemes in the present study, refuting the related null hypothesis. This result can be due to impaired integrity of the framework and reduction in metal volume at the implant sites and being in line with the available literature (Cunha et al. 2008; Ortiz‐Puigpelat et al. 2019; Shahmiri et al. 2014). Thus, the frameworks should be regularly evaluated to prevent complications (Tribst et al. 2020).

The null hypothesis regarding the insignificant effect of the number of implants on metal framework stress in the two occlusal schemes was partially rejected since the placement of two implants decreased the framework stress compared with the conventional RPD and most IARPD models probably due to better stress distribution and further stabilization of the framework.

Implant position had a significant effect on framework stress in both occlusal schemes, refuting the related null hypothesis. IARPD with canine implant showed the highest stress. Ortiz‐Puigpelat et al. (2019) reported that implant placement close to the abutment tooth increased the framework stress. Framework fracture has also been reported following the placement of implants close to the premolar abutment tooth in a clinical study (Verri et al. 2016). The type of occlusal scheme also had a significant effect on framework stress, and generally higher stress was noted in the GF scheme, refuting the related part of the null hypothesis. This finding may be due to the application of a greater load (500N, almost three times the value in CG) from all teeth to the framework. Thus, in the clinical setting, the framework should be strong enough to withstand GF occlusion.

The null hypothesis regarding the insignificant effect of implant placement on resin stress was partially rejected in the GF scheme and completely rejected in the CG since resin stress was higher in IARPD with canine implant than in the conventional RPD with the GF scheme. This finding may be due to resin volume reduction around implant attachment. The effect of the number of implants on resin stress was not significant, confirming the related null hypothesis. The null hypothesis regarding implant position on resin stress was partially rejected in GF and totally rejected in CG scheme since IARPD with canine implant showed the highest resin stress in both GF and CG schemes. In the CG scheme, the lowest stress value was noted in IARPD with the second molar implant. The same results were reported by Ortiz‐Puigpelat et al. (2019). The null hypothesis regarding the insignificant effect of the occlusal scheme on resin stress was partially rejected since higher resin stress was noted in all models in the GF scheme, compared with the CG due to the application of greater occlusal forces as explained earlier.

Stress in the last abutment tooth (lateral incisor) was almost the same in all models, confirming the null hypothesis regarding the insignificant effect of implant placement on abutment tooth stress. This result was in contrast to studies that reported implant placement decreased the abutment tooth stress (Cunha et al. 2008; Eom et al. 2017; Ortiz‐Puigpelat et al. 2019). However, Matsudate et al. (2016) demonstrated greater abutment tooth stress in IARPD with the second molar implant than the conventional RPD. Such conflicting results can be explained by the fact that canine tooth was present in the majority of the aforementioned studies and first or second premolars served as the final abutment tooth while the lateral incisor was the last abutment tooth in the present study, which is smaller and weaker (smaller root area) than premolars. Stress in the abutment tooth was also independent of implant number/position in both occlusal schemes, confirming the related null hypotheses. In contrast, some studies reported that implant placement at the site of second molar increased the abutment tooth stress (Matsudate et al. 2016; Ohyama et al. 2020; Xiao et al. 2014) while some others reported that implant insertion close to the abutment tooth resulted in greater stress generation in the abutment tooth (Cunha et al. 2008; Jia‐Mahasap et al. 2022; Mahshid et al. 2014). Another study (Ohyama et al. 2020) indicated that implant placement close to the abutment tooth increased the bracing effect and decreased the stress and movement of the abutment tooth. It also enabled the elimination of the retentive arm and improved esthetics. Conflicting results can be due to the evaluation of different types of abutment teeth (as explained above). The occlusal scheme had no significant effect on the abutment tooth stress, confirming the related null hypothesis.

The placement of two implants, compared with one, decreased implant stress in both occlusal schemes in the present study, refuting this null hypothesis, and confirming the results of Jia‐Mahasap et al. (2022) Thus, in patients with heavy bite force (as in parafunctional habits), long edentulous ridges, or presence of opposing natural teeth, increasing the number of implants to two or three may decrease implant stress (Jia‐Mahasap et al. 2022). Implant position also had a significant effect on implant stress, refuting the related null hypothesis. The placement of two implants at the sites of the canine and first or second molars decreased stress. In single‐implant RPDs, implant placement at the site of canine yielded the highest implant stress while implant insertion between the first and second premolars resulted in the lowest stress. Previous findings are conflicting in this regard. Ortiz‐Puigpelat et al. (2019) reported higher implant stress in implants placed close to the abutment tooth and lower stress in implants placed at the site of the first molar. Several other studies reported the lowest stress and better stress distribution following implant placement at the site of the first molar (Cunha et al. 2008; Memari et al. 2014; Ortiz‐Puigpelat et al. 2019). A few others showed that implant placement at the site of the premolar tooth increased the flexural torque applied to the implant (Hegazy, Elshahawi, and Elmotayam 2013; Rungsiyakull et al. 2021). To minimize implant stress, the number of implants should be increased and in case of placement of one implant, it should be preferably placed at the inter‐premolar region. In the current study, implant placement at the inter‐premolar region or canine and first molar sites resulted in greater implant stress in the GF than the CG scheme, partially refuting the null hypothesis regarding the insignificant effect of the occlusal scheme on implant stress.

Biomechanically, the results of the present study are consistent with the principles of fixed implant‐supported prostheses. First, the greater the number of implants, the more proportional the stress distribution on them will be. Second, the canine and first molar areas, which are key implant placement areas in implant‐supported fixed prostheses, can also play this key role in implant‐assisted RPDs. Finally, due to the greater retention and stability of the implant‐supported RPDs, the canine guide occlusion, like fixed implant‐supported prostheses, imposes less stress on the implants and prosthetic components.

In the current study, the retention force was not evaluated. Still, studies that evaluated retention force in implant‐assisted RPDs showed that implant placement at the distal extension increases the removal force of RPDs (Rodrigues et al. 2013; Tribst et al. 2020).

This study had some limitations. FEA cannot perfectly simulate the shape, composition, structure, and biological behavior of components in the oral environment. The pattern of jaw resorption was considered almost ideal in the present study, allowing placement of 10 mm implants; however, the situation may be different in the clinical setting. Thus, similar studies are required on the placement of shorter implants. Moreover, considering the strategic role of canine tooth in withstanding forces, future studies are recommended to compare stress patterns in the presence and absence of canine tooth.

## 5 Conclusion

Under the limitations of the current study, the following conclusions were obtained:

1. Dental implant placement decreased the total displacement of IARPDs, increased stress in mechanical components, and had no effect on stress distribution in biological components.
2. Insertion of two instead of one implant decreased implant stress.
3. Implant placement close to the last abutment tooth increased the stress.
4. The GP scheme applied greater stress to mechanical components.

## Author Contributions

Study concept and design: Somayeh Zeighami and Safoura Ghodsi. Acquisition of data: Solmaz Barati. Analysis and interpretation of data: Solmaz Barati, Somayeh Zeighami and Safoura Ghodsi. Drafting of the manuscript: Solmaz Barati, Somayeh Zeighami and Safoura Ghodsi. Critical revision of the manuscript for important intellectual content: Somayeh Zeighami. Statistical analysis: Solmaz Barati. Administrative, technical, and material support: Solmaz Barati, Somayeh Zeighami and Safoura Ghodsi. Study supervision: Somayeh Zeighami and Safoura Ghodsi.

## Conflicts of Interest

The authors declare no conflicts of interest.

## Data Availability

Data are available upon request from the authors.
